# Supplementary material for: Morphodynamics of surface-attached active drops
Source: Nat Commun. 2026 Feb 12;17:1600. doi: 10.1038/s41467-025-68235-w (PMC12905126; doi:10.1038/s41467-025-68235-w)
Supplement: Supplementary file 1 — Supplementary Information [file 41467_2025_68235_MOESM1_ESM.pdf]

Supplementary Information — Morphodynamics of surface-attached active drops

Alejandro Martínez-Calvo [1,2,3,*] and Sujit S. Datta [4,2,†]

[1] Princeton Center for Theoretical Science, Princeton University, New Jersey, 08544, USA
[2] Department of Chemical and Biological Engineering, Princeton University, New Jersey, 08544, USA
[3] Department of Physics, Princeton University, Princeton, NJ 08544, USA
[4] Division of Chemistry and Chemical Engineering, California Institute of Technology, Pasadena, CA 91125, USA

(Dated: February 11, 2026)

Non-dimensionalization. To perform two-dimensional (2D) numerical simulations of the system of equations presented in the main text, we first nondimensionalize it using the characteristic scales also deduced in the main text. To this end, we introduce the following dimensionless variables for the position vector, time, velocity, and pressure fields, respectively,

$$\tilde{\boldsymbol{x}} = \frac{\boldsymbol{x}}{R}, \quad \tilde{t} = \frac{t}{\mu R/\gamma}, \quad \tilde{\boldsymbol{u}} = \frac{\boldsymbol{u}}{\gamma/\mu}, \quad \tilde{\Pi} = \frac{\Pi}{\gamma/R}, \tag{S1}$$

where tildes denote dimensionless variables here in the Supplementary Information. Using the above characteristic scales, the dimensionless volume and momentum conservation equations read, respectively:

$$\boldsymbol{\nabla}\cdot\tilde{\boldsymbol{u}} = 0, \quad \boldsymbol{0} = \boldsymbol{\nabla}\cdot\tilde{\boldsymbol{\sigma}}, \tag{S2a}$$

where $\tilde{\boldsymbol{\sigma}} = -\tilde{\Pi}\boldsymbol{I} + \boldsymbol{\nabla}\tilde{\boldsymbol{u}} + (\boldsymbol{\nabla}\tilde{\boldsymbol{u}})^{\mathrm{T}} - \mathrm{Ca}_\alpha \boldsymbol{pp}$ is the dimensionless stress tensor, and $\mathrm{Ca}_\alpha \equiv \alpha/(\gamma/R)$ is the active Capillary number. Equations (S2) together with the equation for the instantaneous orientation field,

$$\boldsymbol{\nabla}^2\boldsymbol{p} = \boldsymbol{0}, \tag{S3}$$

are a closed system of dimensionless equations that describe the morphodynamics of the surface-attached active drop.

At the liquid-air interface of the drop we impose a kinematic condition specifying that the velocity of the interface is equal to the velocity of the fluid, precluding mass transfer across the interface, the stress balance, and the orientation of the units:

Fluid:

$$(\partial_{\tilde{t}}\tilde{\boldsymbol{r}}_{\mathrm{i}} - \tilde{\boldsymbol{u}})\cdot\hat{\boldsymbol{n}} = 0, \;\; \tilde{\boldsymbol{\sigma}}\cdot\hat{\boldsymbol{n}} = -\tilde{\mathcal{C}}\hat{\boldsymbol{n}} \;\text{ at }\; \tilde{\boldsymbol{r}} = \tilde{\boldsymbol{r}}_{\mathrm{i}}, \tag{S4a}$$

Active units: $\boldsymbol{p} = \boldsymbol{R}_{\mathrm{i}}\cdot\hat{\boldsymbol{t}}$ at $\tilde{\boldsymbol{r}} = \tilde{\boldsymbol{r}}_{\mathrm{i}}$, (S4b)

where $\tilde{\boldsymbol{r}}_{\mathrm{i}}$ is the dimensionless position of the interface, $\hat{\boldsymbol{n}}$ and $\hat{\boldsymbol{t}}$ are the unit normal and tangential vectors to the interface, respectively, and $\tilde{\mathcal{C}} \equiv \boldsymbol{\nabla}\cdot\hat{\boldsymbol{n}}$ is twice the dimensionless mean curvature of the liquid-air interface. Here, $\boldsymbol{R}_{\mathrm{i}}$ is the rotation matrix prescribing the angle θ_{i} that the orientation of the units form with the tangent vector to the interface. For simplicity, we follow Refs. [1, 2] and consider only quarter turns in the orientation angle, i.e., $\theta_{\mathrm{i}} = w_{\mathrm{i}}\pi/2$, where $w \in \mathbb{Z}$ is the winding number (Fig. 1a of the main text).

At the solid substrate, we impose no-permeation and no-slip conditions for the velocity field, i.e., $\tilde{\boldsymbol{u}} = \boldsymbol{0}$ at $\tilde{y} = 0$, and we prescribe the orientation of the active units with respect to the tangent vector to the substrate, equivalently to the condition at the interface, i.e., $\boldsymbol{p} = \boldsymbol{R}_{\mathrm{s}}\cdot\hat{\boldsymbol{e}}_x$, where $\boldsymbol{R}_{\mathrm{s}}$ is the corresponding rotation matrix prescribing the angle θ_{s} at the substrate. For simplicity, we also assume that the orientation angle with the substrate only changes in quarter turns, and thus it is specified by an additional winding number w_{s}.

As initial conditions, we consider the shape of the drop is semicircular and the fluid in the drop is at rest, i.e., $\tilde{\boldsymbol{u}}(\tilde{\boldsymbol{r}}, \tilde{t}=0) = \boldsymbol{0}$ and $\tilde{\Pi}(\tilde{\boldsymbol{r}}, \tilde{t}=0) = 1$.

Describing the nematic field using a nematic tensor. Under certain configurations, describing a nematic field using the vector director free energy in Eq. (3) of the main text is not equivalent to using a nematic $\boldsymbol{Q}$ tensor. The configuration of a surface-attached drop presented in this work is one such case. The key difference between the two descriptions for our surface-attache drop arises from the boundary conditions: in the $\boldsymbol{p}$-based formulation, the imposed boundary conditions do not respect the nematic symmetry ($\boldsymbol{p} \to -\boldsymbol{p}$), causing the active units to become polarized at the substrate and liquid–air interfaces. Here, we compare the morphodynamics of drops described using $\boldsymbol{p}$ with those described using $\boldsymbol{Q}$. To this end, we consider the following free-energy functional for $\boldsymbol{Q}$,

$$F_Q = \int_A \mathrm{d}A - \frac{a}{2}\boldsymbol{Q}:\boldsymbol{Q} + \frac{b}{4}(\boldsymbol{Q}:\boldsymbol{Q})^2 + \frac{K_Q}{2}(\boldsymbol{\nabla}\boldsymbol{Q})^2, \tag{S5}$$

where $\boldsymbol{Q} = S\left(\boldsymbol{pp} - \frac{1}{2}\boldsymbol{I}\right)$, with S a scalar representing the strength of the order parameter, K_Q the Frank elastic constant, and a and b parameters from a Landau expansion in the order parameter that determine the isotropic–nematic transition ($a > 0$ in the nematic phase and $b > 0$ for stability). These parameters define the maximum value the order parameter can take, $S_{\max} = \sqrt{2a/b}$. Here, as in Eq. (S3), we also assume purely relaxational dynamics, i.e.,nematic order is established much more quickly than the flows generated within the drop [3], implying $\boldsymbol{0} = \delta F_Q/\delta\boldsymbol{Q}$, and which yields,

$$\boldsymbol{0} = \left[a - \mathrm{tr}(\boldsymbol{Q}^2)\right]\boldsymbol{Q} + K_Q\boldsymbol{\nabla}^2\boldsymbol{Q}. \tag{S6}$$

The active stress tensor now becomes $\boldsymbol{\sigma}_{\mathrm{a}} = -\alpha\boldsymbol{Q}$, and the total stress tensor reads:

$$\boldsymbol{\sigma} = -\Pi\boldsymbol{I} + \mu\left[\boldsymbol{\nabla}\boldsymbol{u} + (\boldsymbol{\nabla}\boldsymbol{u})^{\mathrm{T}}\right] - \alpha\boldsymbol{Q}. \tag{S7}$$

To compare with the results of the Main Text solving for $\boldsymbol{p}$, we consider both planar and orthogonal anchoring of the nematic with the solid substrate, i.e., $\boldsymbol{Q} = S\left(\hat{\boldsymbol{e}}_x\hat{\boldsymbol{e}}_x - \frac{1}{2}\boldsymbol{I}\right)$ and

$\boldsymbol{Q} = S\left(\hat{\boldsymbol{e}}_y\hat{\boldsymbol{e}}_y - \frac{1}{2}\boldsymbol{I}\right)$, respectively. At the liquid–air interface, we impose either tangential anchoring, $\boldsymbol{Q} = S\left(\hat{\boldsymbol{t}}\hat{\boldsymbol{t}} - \frac{1}{2}\boldsymbol{I}\right)$, or orthogonal anchoring, $\boldsymbol{Q} = S\left(\hat{\boldsymbol{n}}\hat{\boldsymbol{n}} - \frac{1}{2}\boldsymbol{I}\right)$, where $\hat{\boldsymbol{t}}$ and $\hat{\boldsymbol{n}}$ are the unit tangential and normal vectors to the liquid–air interface, respectively.

To make Eq. (S6) dimensionless, we choose $Q_c = \sqrt{a/b}$ as characteristic quantity for the nematic tensor, which yields:

$$\boldsymbol{0} = \left[1 - \mathrm{tr}(\tilde{\boldsymbol{Q}}^2)\right]\tilde{\boldsymbol{Q}} + \tilde{K}_Q\boldsymbol{\nabla}^2\tilde{\boldsymbol{Q}}, \tag{S8}$$

where $\tilde{\boldsymbol{Q}} = \tilde{S}\left(\boldsymbol{pp} - \frac{1}{2}\boldsymbol{I}\right)$ is the dimensionless nematic tensor, $\tilde{K}_Q = (K_Q/a)/R^2$ is a dimensionless parameter that compares the elastic coherence length $\ell_Q = \sqrt{K/a}$ and the drop radius R, and $\tilde{S} = S/\sqrt{a/b}$ is the dimensionless strength of the nematic order parameter. For simplicity, in all simulations we set $\tilde{S} = 1$ at the boundaries, and $\tilde{K}_Q = 1$. Finally, Eq. (S9) reads in dimensionless form:

$$\tilde{\boldsymbol{\sigma}} = -\tilde{\Pi}\boldsymbol{I} + \boldsymbol{\nabla}\tilde{\boldsymbol{u}} + (\boldsymbol{\nabla}\tilde{\boldsymbol{u}})^{\mathrm{T}} - \mathrm{Ca}_\alpha\tilde{\boldsymbol{Q}}, \tag{S9}$$

where, in this case, the active Capillary number reads $\mathrm{Ca}_\alpha \equiv \alpha\sqrt{a/b}/(\gamma/R)$.

Role of nematic advection. To understand the effect of nematic advection, we consider the complete dynamical equation for $\boldsymbol{Q}$, which in dimensionless form reads:

$$\tilde{\Gamma}_Q\left[\partial_{\tilde{t}}\tilde{\boldsymbol{Q}} + \tilde{\boldsymbol{u}}\cdot\boldsymbol{\nabla}\tilde{\boldsymbol{Q}} - (\tilde{\boldsymbol{Q}}\cdot\tilde{\boldsymbol{\Omega}} - \tilde{\boldsymbol{\Omega}}\cdot\tilde{\boldsymbol{Q}})\right] = \left[1 - \mathrm{tr}(\tilde{\boldsymbol{Q}}^2)\right]\tilde{\boldsymbol{Q}} + \tilde{K}_Q\boldsymbol{\nabla}^2\tilde{\boldsymbol{Q}}, \tag{S10}$$

where $2\tilde{\boldsymbol{\Omega}} = \boldsymbol{\nabla}\tilde{\boldsymbol{u}} - (\boldsymbol{\nabla}\tilde{\boldsymbol{u}})^{\mathrm{T}}$ is the dimensionless vorticity tensor, and $\tilde{\Gamma}_Q = (\Gamma_Q/\mu)\gamma/(aR)$ is a dimensionless parameter that compares the nematic rotational viscosity Γ_Q with the liquid viscosity μ.

We also consider the nematic advection when the nematic is described by the director field $\boldsymbol{p}$. The dynamical equation for $\boldsymbol{p}$ [Eq. (S3)] now reads:

$$\tilde{\Gamma}\left(\partial_{\tilde{t}}\boldsymbol{p} + \tilde{\boldsymbol{u}}\cdot\boldsymbol{\nabla}\boldsymbol{p} + \tilde{\boldsymbol{\Omega}}\cdot\boldsymbol{p}\right) = \boldsymbol{\nabla}^2\boldsymbol{p}, \tag{S11}$$

where $\tilde{\Gamma} \equiv (\Gamma R^2/K)/t_c = (\Gamma R^2/K)/(\mu R/\gamma)$, is a dimensionless parameter that compares the characteristic elastic relaxational time scale of the nematic with the viscous characteristic time scale of the liquid $t_c = \mu R/\gamma$.

Elastic stresses. In the main text, we neglect the elastic stresses exerted by the active units, whose contribution to the total dimensionless stress tensor reads

$$\tilde{\boldsymbol{\sigma}}_e = \frac{\tilde{E}}{2}|\boldsymbol{\nabla}\boldsymbol{p}|^2\boldsymbol{I} - \tilde{E}\boldsymbol{\nabla}\boldsymbol{p}\cdot\boldsymbol{\nabla}\boldsymbol{p}^{\mathrm{T}}, \tag{S12}$$

where $\tilde{E} = K/(\gamma R)$ is a dimensionless parameter comparing nematic elastic forces with capillary forces. Thus, to neglect both elastic stresses and advective effects (i.e., for $\tilde{E} \ll 1$ and $\tilde{\Gamma} \ll 1$) we require $\Gamma/\mu \ll \tilde{E}$. This approximation is appropriate for viscous droplets with a large surface tension coefficient relative to the nematic elastic modulus. Typical values of the elastic constant are $K = 10^{-12}$–10^{-11} N. Considering typical surface tension coefficients in the range $\gamma = 10^{-2}$–10^{-1} N m^{-1} and small droplet radii in the range $R = 10^{-6}$–10^{-3} m (so that the Stokes limit applies), we obtain $\tilde{E} = 10^{-4}$–10^{-7}, indicating that neglecting elastic stresses is a reasonable approximation. It is likely that only in small regions—such as near the contact line—elastic stresses play a significant role.

Substrate friction. We explore the drop dynamics when it is not pinned to the solid substrate. To this end, we introduce a finite slip length ℓ_s between the active liquid and the substrate accounting for substrate friction [4, 5]:

$$\boldsymbol{u} = \ell_s\hat{\boldsymbol{e}}_y\cdot\left[\boldsymbol{\nabla}\boldsymbol{u} + (\boldsymbol{\nabla}\boldsymbol{u})^{\mathrm{T}}\right]\cdot(\boldsymbol{I} - \hat{\boldsymbol{e}}_u\hat{\boldsymbol{e}}_y) = \ell_s\partial_y u\hat{\boldsymbol{e}}_x, \tag{S13}$$

where ℓ_s denotes the local slip length. In dimensionless form, Eq. (S13) reads:

$$\tilde{\boldsymbol{u}} = \tilde{\ell}_s\hat{\boldsymbol{e}}_y\cdot\left[\boldsymbol{\nabla}\tilde{\boldsymbol{u}} + (\boldsymbol{\nabla}\tilde{\boldsymbol{u}})^{\mathrm{T}}\right]\cdot(\boldsymbol{I} - \hat{\boldsymbol{e}}_u\hat{\boldsymbol{e}}_y) = \tilde{\ell}_s\partial_{\tilde{y}}\tilde{u}\hat{\boldsymbol{e}}_x, \tag{S14}$$

where $\tilde{\ell}_s \equiv \ell_s/R$ is the dimensionless slip length. We perform numerical simulations incorporating Eq. (S14) boundary condition for a representative case in which the nematic is described by the director field $\boldsymbol{p}$, and where $(w_s, w_i) = (0, 1)$ and $\mathrm{Ca}_\alpha = 4.5$. As expected, the drop can migrate *only* when symmetry is broken, and it does so in a treadmilling fashion (Fig. S5) [1]

Numerical simulations. We carry out numerical simulations of Eqs. (S2)- (S4) using the finite-element method. To this end, all the dimensionless equations are written in weak form by means of the corresponding integral scalar product, defined in terms of test functions for the pressure $\tilde{\Pi}(\tilde{\boldsymbol{r}}, \tilde{t})$, the velocity $\tilde{\boldsymbol{u}}(\tilde{\boldsymbol{r}}, \tilde{t})$, and the orientation of active units $\tilde{\boldsymbol{p}}(\tilde{\boldsymbol{r}}, \tilde{t})$. By using Green identities we obtain an integral bilinear system of equations for the set of variables and their corresponding test functions:

$$\int_{\tilde{A}} \mathrm{d}\tilde{A}\, \boldsymbol{\nabla}\boldsymbol{p} : \boldsymbol{\nabla}\tilde{\boldsymbol{\phi}}_p = 0, \tag{S15a}$$

$$\int_{\tilde{A}} \mathrm{d}\tilde{A}\, \tilde{\phi}_\Pi(\boldsymbol{\nabla}\cdot\tilde{\boldsymbol{u}}) = 0, \tag{S15b}$$

$$\int_{\tilde{A}} \mathrm{d}\tilde{A}\, \tilde{\boldsymbol{\sigma}} : \boldsymbol{\nabla}\tilde{\boldsymbol{\phi}}_u - \int_{\tilde{\ell}} \mathrm{d}\tilde{\ell}\, \tilde{\boldsymbol{\sigma}}\cdot\hat{\boldsymbol{n}}\cdot\tilde{\boldsymbol{\phi}}_{\boldsymbol{u}} = 0, \tag{S15c}$$

where $\tilde{\phi}_{\boldsymbol{p}}$, $\tilde{\phi}_\Pi$, and $\tilde{\phi}_{\boldsymbol{u}}$ are the test functions for the orientation, pressure, and velocity fields, respectively. Here, $\tilde{A}$ is the dimensionless area of the drop and $\tilde{\ell}$ the dimensionless length of the boundaries, where $\mathrm{d}\tilde{A}$ and $\mathrm{d}\tilde{\ell}$ are the corresponding surface and line elements, respectively. In Eq. (S15b) we impose the Dirichlet boundary conditions as pointwise constraints on the boundaries for $\boldsymbol{p}$, specifying the angle of the active units with the substrate and deformable interface. Similarly, we impose the no-flux and no-slip boundary conditions

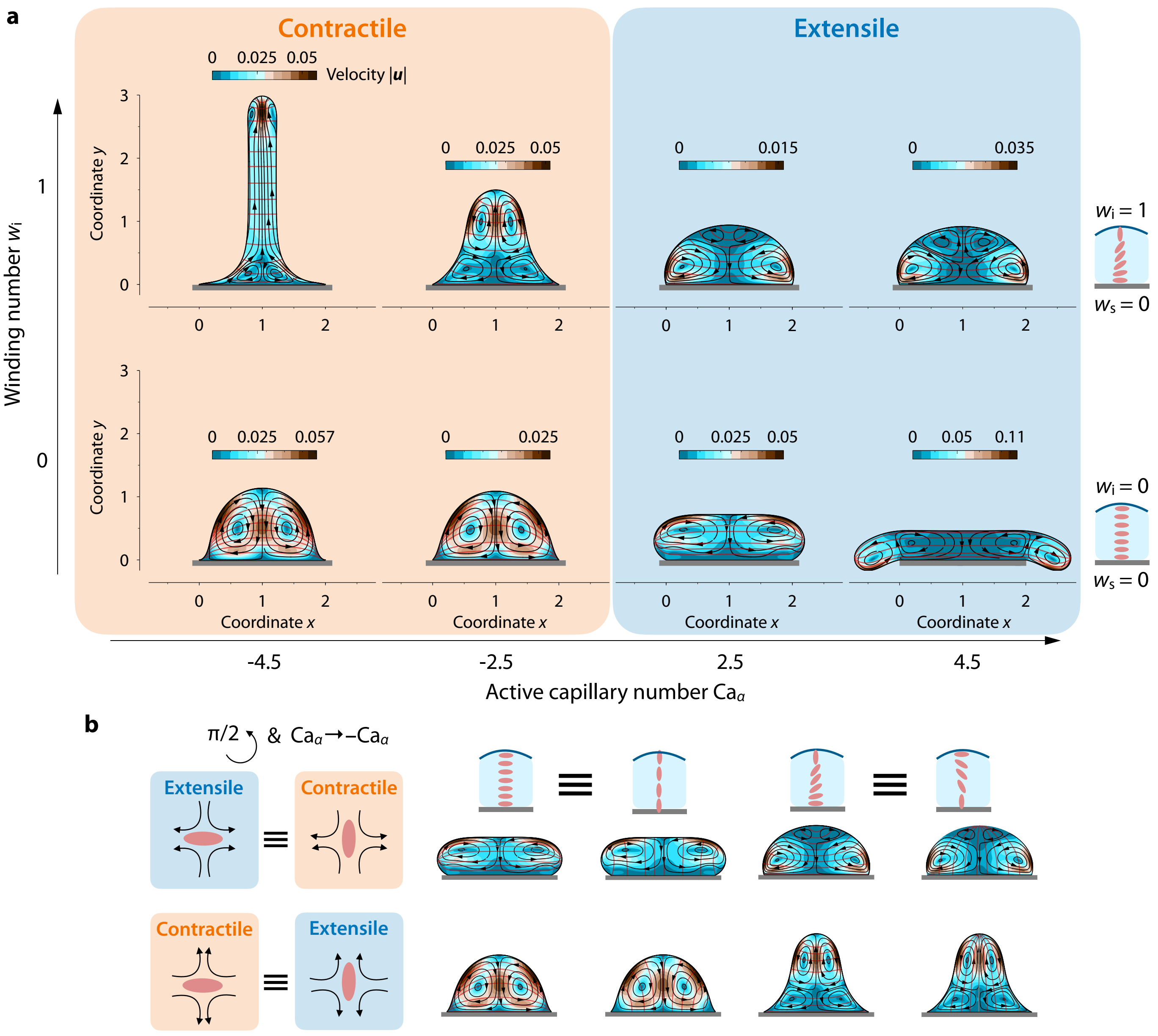

Fig. S1. **Surface-attached active nematic drops also exhibit a rich array of equilibrium shapes and flows when described using the nematic tensor $\boldsymbol{Q}$.** **a**, Steady-state shapes and self-generated flows of a 2D active nematic drop attached to a rigid, planar substrate, as a function of the active capillary number Ca_α and the interfacial winding number w_i, for planar substrate anchoring, $w_\mathrm{s} = 0$, $\tilde{K}_Q = 1$, and $\tilde{S} = 1$. In this case, where the nematic field is described by the nematic tensor $\boldsymbol{Q}$, $w_\mathrm{i} = 2$ is identical to $w_\mathrm{i} = 0$. The color plots display the magnitude of the flow velocity $|\boldsymbol{u}|$ inside the drop. The black curves represent the flow streamlines, and the red curves depict the orientation of the active units. **b**, The steady states of a surface-attached active drop for planar ($w_\mathrm{s} = 0$) and orthogonal ($w_\mathrm{s} = 1$) unit anchoring with the substrate are equivalent under the transformations $w \to w + \pi/2$ and $\mathrm{Ca}_\alpha \to -\mathrm{Ca}_\alpha$. Here, $|\mathrm{Ca}_\alpha| = 2.5$.

for $\tilde{\boldsymbol{u}}$ on the substrate. To impose the stress balance at the deformable interface, the second term of Eq. (S15c) is expressed as: $\int_{\tilde{\ell}} \mathrm{d}\tilde{\ell}\, \boldsymbol{\nabla}_\mathrm{s} \cdot \tilde{\boldsymbol{\phi}}_{\boldsymbol{u}}$, where $\boldsymbol{\nabla}_\mathrm{s} = (\boldsymbol{I} - \hat{\boldsymbol{n}}\hat{\boldsymbol{n}}) \cdot \boldsymbol{\nabla}$ is the surface gradient operator.

Equations (S15) are discretized using Taylor-Hood triangular elements for pressure and velocity and their corresponding test functions, ensuring numerical stability, and second-order Lagrange polynomials for the orientation field and its test function. To account for the deformation of the interface, we use the arbitrary Lagrangian-Eulerian technique, which allows us to track the interface imposing the kinematic boundary condition by prescribing the normal velocity of the mesh elements along the interface. In particular, the displacement of the mesh elements is computed by solving the Laplace equation for the displacement field, i.e., $\boldsymbol{\nabla}^2 \tilde{\boldsymbol{q}} = \boldsymbol{0}$. Regarding the time-stepping, we employ a 4th-order variable-step BDF

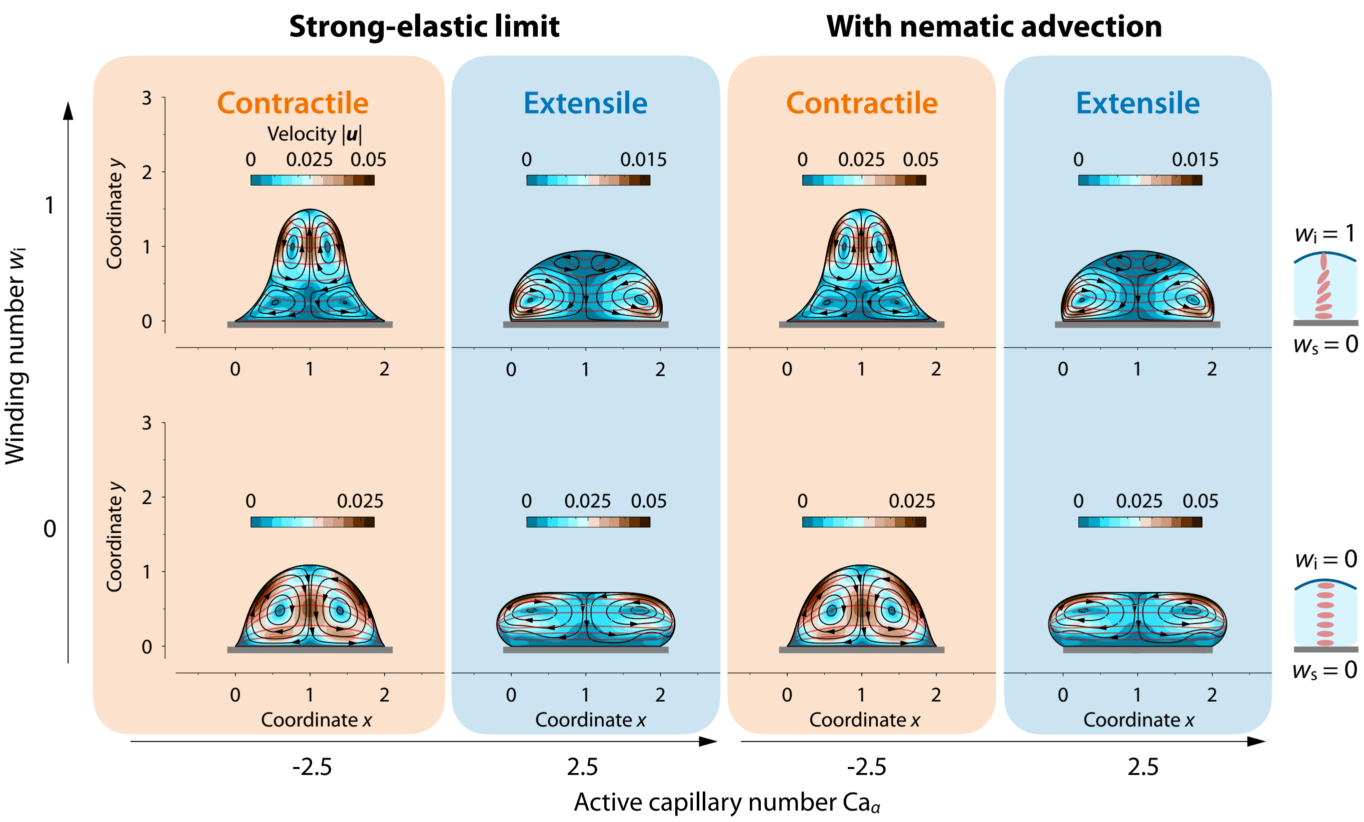

Fig. S2. **Advection of active units does not affect the steady-state shapes and flows of the drop**. Steady-state shapes and self-generated flows of the surface-attached active drop for $w_s = 0$ comparing the case without advection of active units (i.e., the strong elastic limit, $\tilde{\Gamma}_Q = 0$; left columns), to the case with advection included ($\tilde{\Gamma}_Q = 1$; right columns). In both cases, we consider all combinations of $w_i = (0, 1)$ and $Ca_\alpha = (-2.5, 2.5)$. Here, the nematic field is described by the nematic tensor $\boldsymbol{Q}$.

method. The tolerance of the nonlinear method is always set below 10^{-6}. The time-dependent solver was complemented with an automatic remeshing algorithm which generates a new mesh when the triangular elements become significantly distorted due to large deformations of the drop. Using this approach, we are able to fully resolve large deformations and the emergence of intricate interfacial morphologies, as well as thin films near the drop contact line (see Fig. S4).

* amcalvo@princeton.edu

† ssdatta@caltech.edu

[1] A. Loisy, J. Eggers, and T. B. Liverpool, "Tractionless self-propulsion of active drops," Phys. Rev. Lett. **123**, 248006 (2019).
[2] A. Loisy, J. Eggers, and T. B. Liverpool, "How many ways a cell can move: the modes of self-propulsion of an active drop," Soft Matter **16**, 3106–3124 (2020).
[3] S. Shankar, S. Ramaswamy, M.C. Marchetti, and M.J. Bowick, "Defect unbinding in active nematics," Phys. Rev. Lett. **121**, 108002 (2018).
[4] Eric Lauga and Howard A Stone, "Effective slip in pressure-driven stokes flow," J. Fluid Mech. **489**, 55–77 (2003).
[5] A. Martínez-Calvo, D. Moreno-Boza, and A. Sevilla, "The effect of wall slip on the dewetting of ultrathin films on solid substrates: Linear instability and second-order lubrication theory," Phys. Fluids **32** (2020).

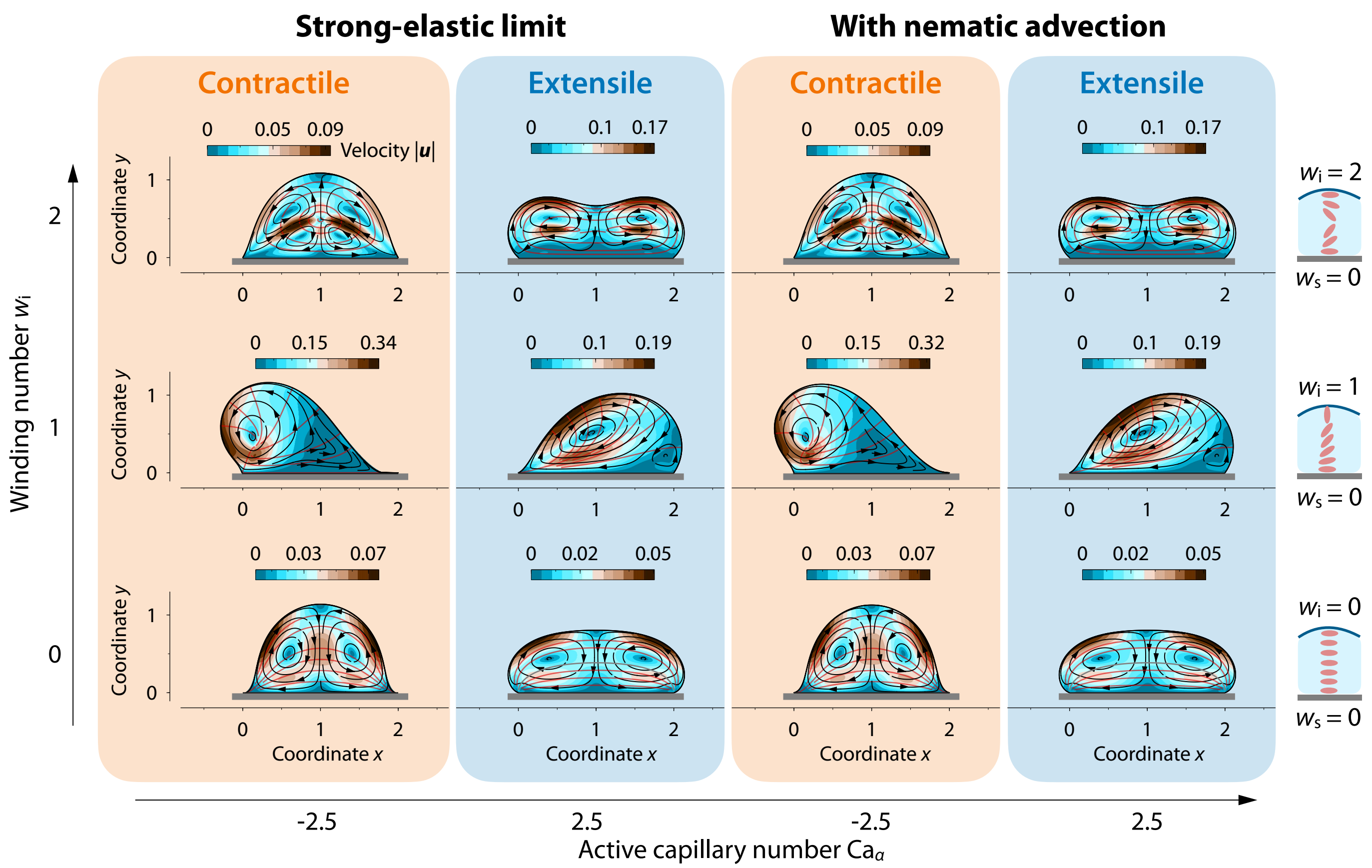

Fig. S3. Advection of active units does not affect the steady-state shapes and flows of the drop. Same as in Fig. S2 except the nematic field is described by the orientation vector field $\boldsymbol{p}$.

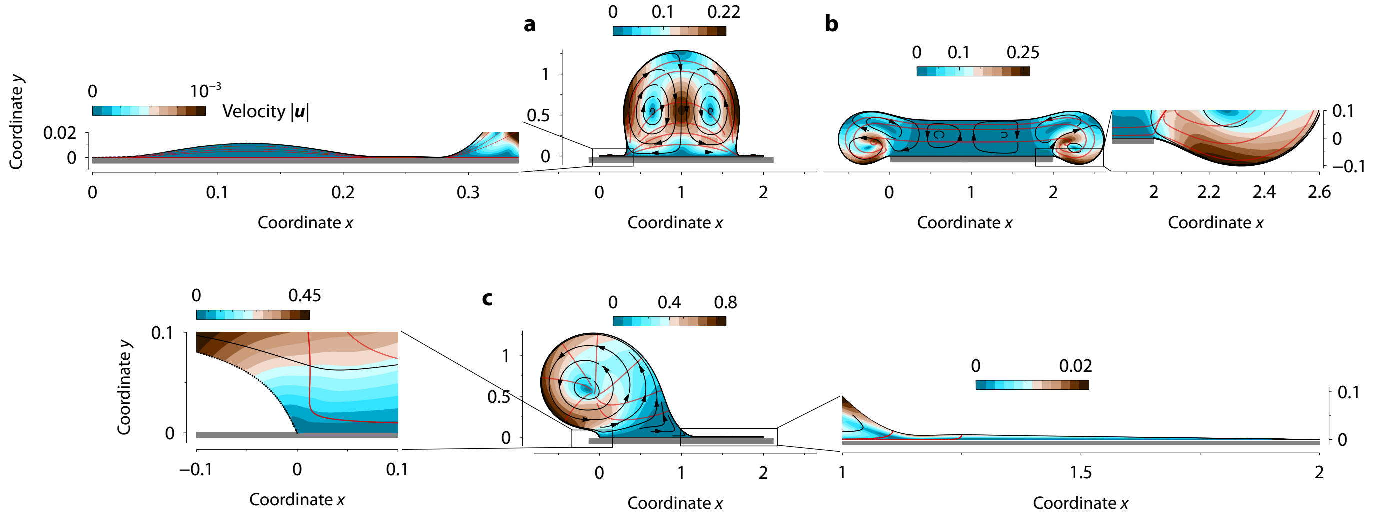

Fig. S4. Zoomed-in view of triple contact points. Zoomed-in view of triple contact points for $w_s = 0$ and **a**, $w_i = 0$ and $Ca_\alpha = -4.5$; **b**, $w_i = 0$ and $Ca_\alpha = 4.5$; and **c**, $w_i = 1$ and $Ca_\alpha = -4.5$.

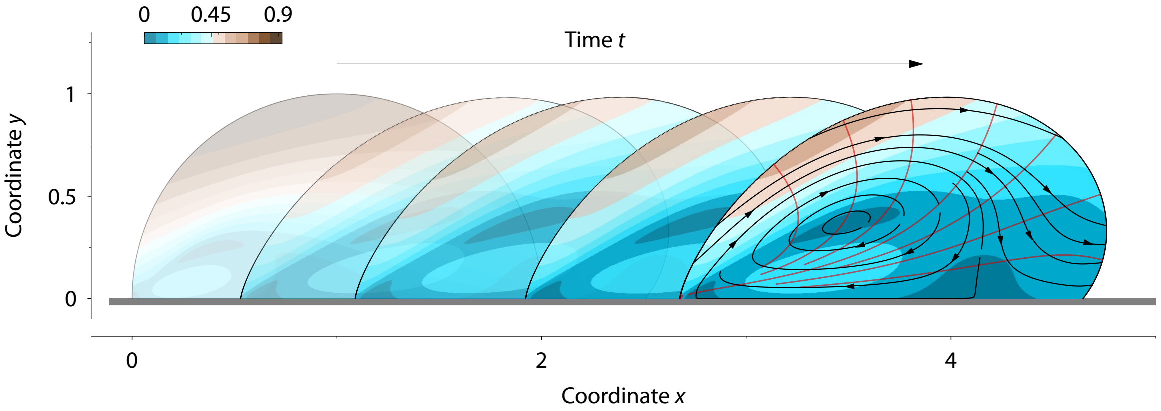

Fig. S5. **Symmetry-breaking drops with finite substrate slip can migrate.** An active nematic drop with $w_\mathrm{s} = 0$, $w_\mathrm{i} = 1$, and $\mathrm{Ca}_\alpha = 4.5$, breaks symmetry and migrates along the solid substrate when finite friction slip is allowed ($\tilde{\ell}_\mathrm{s} = 0.1$).
